# RETRACTED: Dai et al. Decreased Expression of CIRP Induced by Therapeutic Hypothermia Correlates with Reduced Early Brain Injury After Subarachnoid Hemorrhage. *J. Clin. Med.* 2022, *11*, 3411

**DOI:** 10.3390/jcm15207969

**Published:** 2026-10-15

**Authors:** Haibin Dai, Yan Zhou, Yue Lu, Xiangsheng Zhang, Zong Zhuang, Yongyue Gao, Guangjie Liu, Chunlei Chen, Jin Ma, Wei Li, Chunhua Hang

**Affiliations:** 1 Department of Neurosurgery, Nanjing Drum Tower Hospital, Medical School of Nanjing University, Nanjing 210008, China; daisea2006@sina.com (H.D.); oatmeal0329@163.com (Y.Z.); njuluyue@163.com (Y.L.); zhuangzong@126.com (Z.Z.); tallergao@163.com (Y.G.); 15195880897@163.com (G.L.); 15720801655@163.com (C.C.); lwxzlw@126.com (W.L.); 2 Department of Neurosurgery, Beijing Friendship Hospital, Capital Medical University, Beijing 100050, China; 15895971847@163.com; 3 Department of Medical Equipment, School of Aerospace Medicine, Air Force Medical University, Xi’an 710032, China

The journal retracts the article titled “Decreased expression of CIRP induced by therapeutic hypothermia correlates with reduced early brain injury after subarachnoid hemorrhage” [1], cited above.

Following publication, concerns were brought to the attention of the Editorial Office regarding the integrity of images presented in this publication [1].

In accordance with the journal’s complaint procedure, an investigation was conducted by the Editorial Office and Editorial Board. The investigation identified evidence of inappropriate image editing in Figures 2 and 5 [1]. In addition, the Editorial Board was unable to fully verify the authenticity of the images presented in Figures 4 and 5 [1]. Although the authors cooperated with the investigation, the explanations and supporting materials provided were not deemed sufficient by the Editorial Board to resolve the identified concerns. Consequently, the Editorial Board has lost confidence in the reliability of the findings and decided to retract this publication [1], as per MDPI’s retraction policy.

This retraction was approved by the Editor-in-Chief of the *Journal of Clinical Medicine*.

The authors have been informed of this retraction and have indicated their disagreement with this decision.
